# Shrinkage Compensation and Effect of Building Orientation on Mechanical Properties of Ceramic Stereolithography Parts

**DOI:** 10.3390/polym15193877

**Published:** 2023-09-25

**Authors:** Piyush Arora, Khaled G. Mostafa, Emmanuel Russell, Shirin Dehgahi, Sajid Ullah Butt, Didier Talamona, Ahmed Jawad Qureshi

**Affiliations:** 1 Additive Design and Manufacturing Systems Laboratory, Department of Mechanical Engineering, University of Alberta, Edmonton, AB T6G 2R3, Canada; parora2@ualberta.ca (P.A.); kmostafa@ualberta.ca (K.G.M.); dehgahi@ualberta.ca (S.D.); sajidullahbutt@ceme.nust.edu.pk (S.U.B.); ajqureshi@ualberta.ca (A.J.Q.); 2 Fraunhofer Institute of Production Technology IPT, RWTH Aachen, 52062 Aachen, Germany; emmanuel.russell@rwth-aachen.de; 3 Department of Mechanical Engineering (CEME), National University of Sciences and Technology (NUST), Islamabad 46000, Pakistan; 4 School of Engineering and Digital Sciences, Nazarbayev University, Astana 010000, Kazakhstan

**Keywords:** ceramics, additive manufacturing, vat polymerization, shrinkage, porosity, mechanical properties, ceramic stereolithography

## Abstract

Stereolithography additive manufacturing (SLA-AM) can be used to produce ceramic structures by selectively curing a photosensitive resin that has ceramic powder in it. The photosensitive resin acts as a ceramic powder binder, which is burned, and the remaining ceramic part is sintered during post-processing using a temperature–time-controlled furnace. Due to this process, the ceramic part shrinks and becomes porous. Moreover, additive manufacturing leads to the orthotropic behavior of the manufactured parts. This article studies the effect of the manufacturing orientation of ceramic parts produced via SLA-AM on dimensional accuracy. Scaled CAD models were created by including the calculated shrinkage factor. The dimensions of the final sintered specimens were very close to the desired dimensions. As sintering induces porosity and reduces the mechanical strength, in this study, the effect of orientation on strength was investigated, and it was concluded that the on-edge specimen possessed by far the highest strength in terms of both compression and tension.

## 1. Introduction

Ceramics possess a high strength-to-weight ratio [1,2] and excessive wear and corrosion resistance [2] and can withstand high temperatures [3,4]. For these reasons, ceramics have been used in the automotive, aerospace, medical, defense, and other sectors. Due to the brittleness and hardness of ceramics, machining is a cumbersome task [1,2,5,6]. Additive manufacturing (AM) makes it feasible to produce ceramic components with complex geometries [7,8,9]. AM technologies used to produce ceramic parts include selective laser sintering (SLS), binder jetting, vat polymerization, direct ink writing (DIW), and fused filament fabrication (FFF) [10,11,12].

Selective laser sintering (SLS) technology sinters the pre-heated powder layer in a closed inert chamber using a high-powered pulsed laser [2]. Since ceramics possess a high melting temperature, it is infeasible to fuse their powder. Consequently, ceramic components produced through SLS exhibit high porosity and a theoretically low density of about 85% [13]. In order to solve this drawback, an optimal composition of low-melting binder can be added to the ceramic powder to assist in densification. This requires pyrolysis of the polymeric binder, leading to significant part shrinkage. Manufacturing ceramics using SLS induces high thermal gradients, causing residual stress [13,14] and resulting in crack formation [2]. Zheng et al. [15] improved the mechanical characteristics of silica ceramics by implementing vacuum infiltration and adding mullite fibers to silica cores. The flexural strength increased from 4 MPa to 7.45 MPa. Upon increasing the temperature to 1550 °C, the flexural strength reached 15.04 MPa.

In binder jetting, ceramics are manufactured by injecting a liquid binder through a print head and selectively depositing it on the ceramic powder to bind it and procure green ceramic parts. The strength and density of the green components are very low. A de-binding process follows, and the parts are sintered. Binder jetting is considerably cheaper than SLS printing; however, it produces weaker parts [16]. By fine-tuning the process parameters, i.e., powder size, layer thickness, and sintering profile, the compression strength was increased by 82.98% in one study when the sintering dwell time was ramped up from 2 to 16 h, and the elastic modulus increased by 73% [17,18]. Decreasing the powder size increased the density of the produced parts and decreased the shrinkage. The shrinkage was estimated to be 8% in the layer plane direction and about 10% in the printing direction [18].

Vat photopolymerization technology involves filling photocurable resins with ceramic particles; the resin is then selectively polymerized with exposure to UV light. As a result, the ceramic particles are bonded by the polymerized resin. The produced green parts have very low strength. The green parts are then placed in an oven programmed with a specific temperature–time profile to ensure that the pyrolysis of the polymer occurs, and the ceramic particles are sintered. The thermal treatment of the green parts leads to warpage, porosity, cracks, and shrinkage, which have an impact on the manufactured ceramics’ mechanical properties and dimensional accuracy [19]. The reported flexural strength of silica ceramics produced via SLA is about 13 MPa, and the density of the sintered final parts is 1.57 g/cm^3^ [20].

Additive manufacturing is a layer-by-layer technique; therefore, the mechanical properties, geometrical accuracy, and shrinkage ratio highly depend on the manufacturing orientation. This also influences the location and orientation of cracks in the sintered silica ceramic specimens [21]. The linear shrinkage ratio is also a function of the printing direction [22,23]. The shrinkage value affects the final dimensional accuracy of the part. If the shrinkage factor is known, it can be used to produce enlarged parts to reduce the shrinkage effect. 

In this study, stereolithography-based additively manufactured silica ceramic parts were manufactured, shrinkage percentages in each manufacturing direction were identified, and compensation factors were integrated into CAD models to produce larger green parts. The proposed approach was validated by comparing the sintered part with the required dimensions. Moreover, the influence of the build orientation on the compressive and tensile strengths of the sintered parts was investigated. Porosity and crack growth were also studied using computed tomography (CT) scanning.

## 2. Materials and Methods

This section describes the manufacturing tools, material, design of the specimens, calculation of the shrinkage method, and the pre- and post-processing steps used in this project. The methods used to analyze the results and the recorded data are also detailed here.

### 2.1. Manufacturing Platform

A desktop 3D printer, Form 2 by Formlabs Somerville, MA, USA, was used as the additive manufacturing platform. It is a stereolithography (SLA) vat photopolymerization technology. Form 2 has a laser spot size of 140 µm, and its layer thickness ranges between 25 and 100 µm depending on the photopolymer resin type. The machine’s manufacturing envelope is 145 mm × 145 mm × 175 mm (L × W × H). For more information on the printing process, refer to the study by Garcia et al. [24].

### 2.2. Material

In this work, silica-based ceramic resin was used—specifically, the original equipment manufacturer (OEM) proprietary ceramic resin produced by Formlabs, USA. According to the OEM, the fired part should have a tensile modulus of 50 GPa, compression strength of 72 MPa, a Poisson’s ratio of 0.14, and density of 1.9 g/cm^3^ [25,26].

### 2.3. Specimen Design

**Figure 1:**
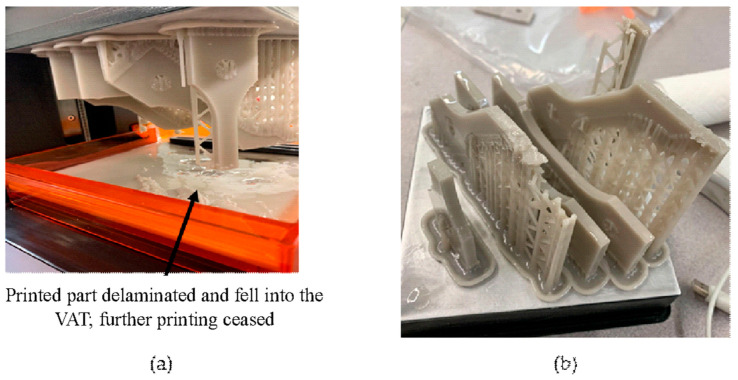
(**a**,**b**) Failure of the original Ford design during manufacturing.

**Figure 2:**
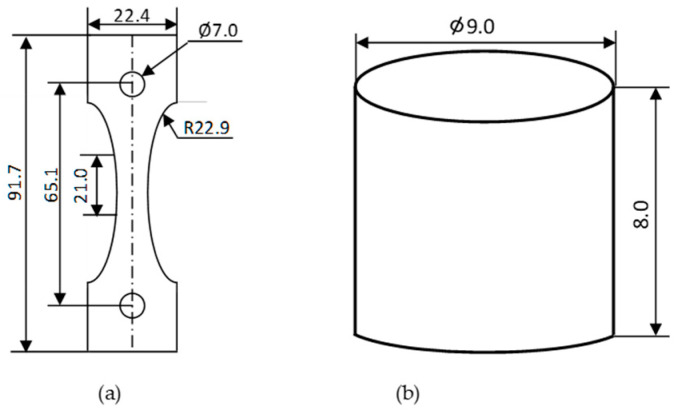
(**a**) Modified Ford tensile test specimen from ISO 15490 [27] and (**b**) compression test specimen adapted from ISO 17162 [28] (all dimensions are in mm).

Various test specimens were used to evaluate the tension and compression characteristics. For the tensile test, a Ford specimen was selected based on ISO 15490 [27]. However, due to its relatively lower thickness, it consistently failed during manufacturing, as shown in Figure 1. To overcome this issue, the width and the height of the Ford tensile specimen were scaled down by 20% and 30%, respectively, while the thickness was kept unchanged, as shown in Figure 2a. The specimen’s final gauge length was 21 mm and the gauge cross-section was 7 mm × 5 mm (width × thickness). For the compression test, the specimen was designed according to ISO 17162 [28], as shown in Figure 2b.

### 2.4. Shrinkage Factor Calculation Strategy

**Figure 3:**
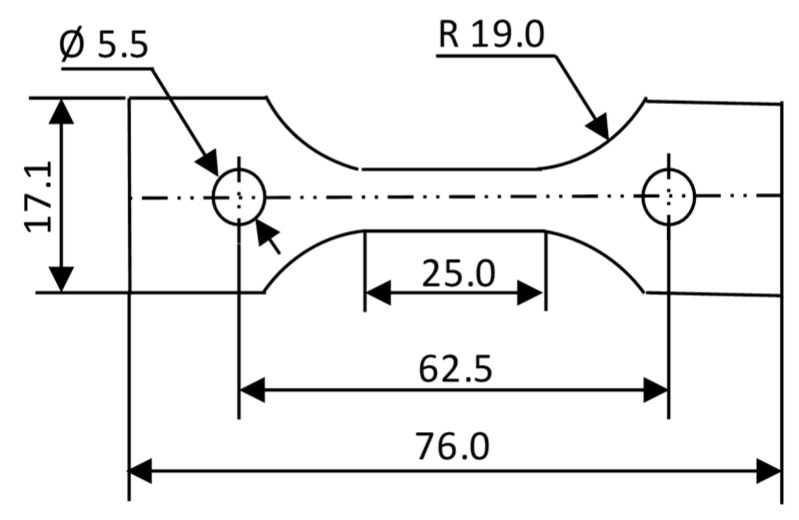
Dimensions of Norton’s tensile specimen (adapted from ISO 17162) manufactured and sintered to estimate the compensation factor (all dimensions are in mm).

To calculate the shrinkage compensation factor experienced by the specimen after the sintering, a pilot experimental analysis was set up. For this purpose, the Norton design for the tensile ceramic specimen was selected from the ISO-15490 [27], as shown in Figure 3. This specimen was printed in three orientations (on-edge, inclined, and vertical) and sintered. The dimensions were measured, and the shrinkage percentage was calculated for all the dimensions of interest using the formula depicted in Equation (1).

(1) $$Shrinkage\;percent=\left(\frac{CAD\;model\;dimension-Sintered\;dimension}{CAD\;model\;dimension}\right)\times 100,$$

### 2.5. Pre-Processing

**Figure 4:**
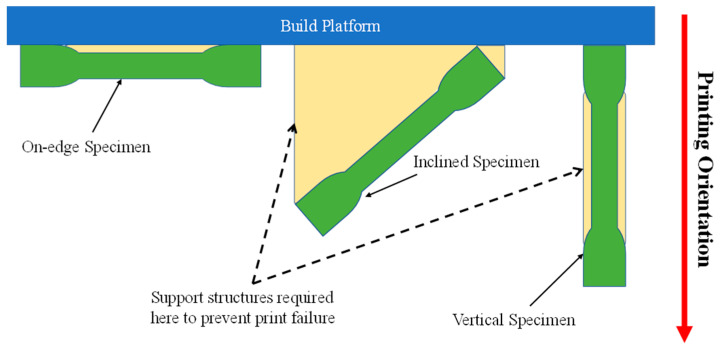
Layout of different printing orientations of the test specimens.

Due to the material’s orthotropic behavior, introduced by additive manufacturing, the mechanical properties were expected to vary in the three orthogonal axes. Therefore, each test specimen was manufactured in three different orientations, parallel to the horizontal plane (on-edge), inclined by 45° on the horizontal plane (inclined), and perpendicular to the horizontal plane (vertical), as shown in Figure 4.

The specimens were pre-processed using the Preform 3.23.0 software and five replicates were manufactured for each orientation, bringing the total manufactured specimens to 15 for each of the tensile and compression tests. To minimize warpage during manufacturing and to ensure that the specimens did not separate from the build platform, supports and mini rafts were added. The layer thickness for all the specimens was set to 100 µm.

### 2.6. Post-Processing

Post-processing had four main steps.

**Figure 5:**
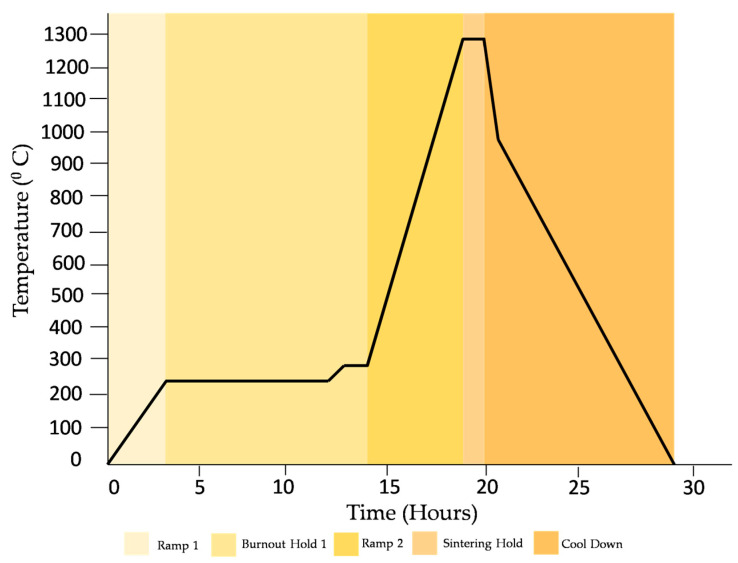
Burnout and sintering schedule provided by Formlabs [25,26].

1. Washing—The manufactured specimens were rinsed with isopropyl alcohol for six minutes to ensure the removal of any unpolymerized resin.
2. Post-curing—Each side was kept under UV light for 30 min, with a total of 60 min for the whole part. This step ensured that the specimens could sustain the handling during the remaining post-processing steps.
3. Finishing—The supports were removed and the surfaces were sanded with fine sandpaper.
4. Sintering—The specimens were fired in a furnace programed with a specific temperature–time profile, as shown in Figure 5. The burnout hold time is related to the thickness of the specimens. During this period, the polymer matrix decomposes and evaporates. This burnout and sintering cycle was chosen according to the ceramic resin user manual provided by Formlabs [25,26]. To avoid structural cracking, the cooldown rate was set to 2 °C/min. The sintering was performed in a 1710 FL furnace developed by CM furnaces, with working dimensions of 10 in × 10 in × 10 in. Each specimen was sintered individually and placed in the center of the furnace.

### 2.7. Dimensions and Weight Measurements

**Figure 6:**
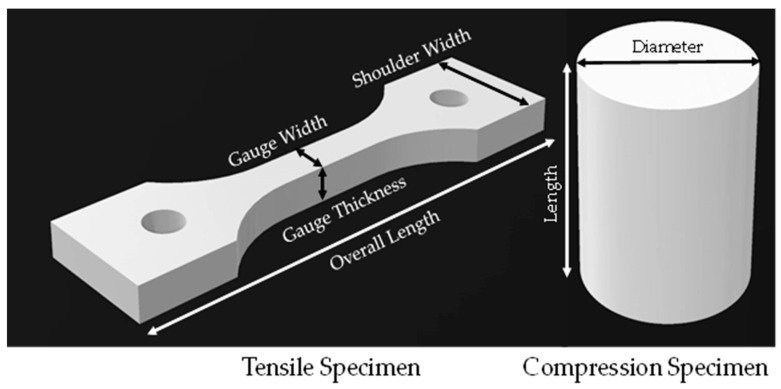
Measured dimensions for each test specimen.

To measure the dimensions of the specimen after manufacturing and post-curing, a digital caliper with 0.1 mm resolution was used and the results were compared to the nominal compensated dimensions in the CAD file. In addition, a second comparison was performed between the dimensions of the sintered specimen and the desired value to be achieved, which is discussed in Section 3.3. Four dimensions of the tension samples and two dimensions of the compression samples were measured, as shown in Figure 6. Each dimension was measured three times at three different locations for all the specimens to calculate an average value, as recommended in ISO-15490 [27].

To compare the weights of the same specimens printed in different manufacturing orientations and to compare the variations in the weights of all specimens due to the sintering process, an electronic weighing scale with accuracy of 0.01 g was used.

### 2.8. Computed Topography (CT) Scan

The microstructure of the specimen significantly impacts the strength of the material. Some compression specimens were inspected for porosity and the void percentage before and after the sintering process by means of a computed tomography (CT) scan through a ZEISS Xradia Versa 620 X-ray Microscope (XRM, Oberkochen, Germany). The machine detector had 2048 × 2048 pixels and the compression specimen was 9mm long; hence, it was decided to adopt a feature size of 5 microns. The layer thickness of each print was 100 microns, so a feature size of 5 microns facilitated a more accurate analysis of the specimens than a larger feature size. 

### 2.9. Mechanical Testing

The machine used for tensile and compression tests was the 3360 series universal testing system from Instron, Norwood, MA, USA. Displacement control mode was used, in which the respective specimens were tensioned and compressed at a rate of 0.5 mm/min in both tests. The parameters evaluated for each specimen were ultimate strength, ultimate strain, yield stress, and yield strain. All the aforementioned material characteristics were obtained in accordance with the methods described in ISO 527-1 [29] and 527-2 [30]. Since there were significant cracks in the sintered specimens, it was a challenge to incorporate strain sensors. Therefore, strain was measured directly from the distance traveled by the machine.

**Figure 7:**
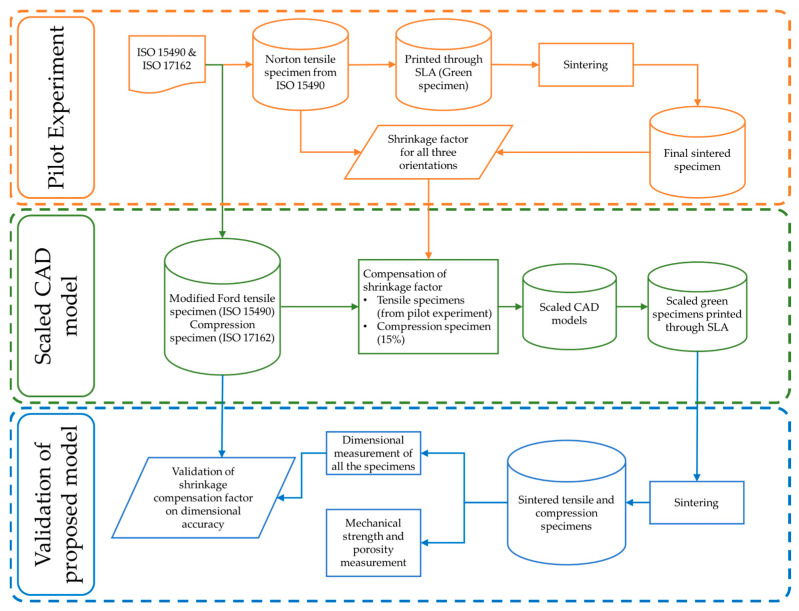
Flowchart of the proposed methodology.

Ultimate strength is the maximum stress; the corresponding strain is the ultimate strain. For yield strength, the slope was calculated for the full range, and when the slope was negative for the first time, the previous stress value was realized as the yield strength and its corresponding strain as the yield strain. Figure 7 shows the flow chart of the proposed methodology.

## 3. Results and Discussion

The manufactured parts were inspected visually for cracks and warpage. The accuracy of the shrinkage compensation factor was assessed and a dimensional analysis was performed, as detailed in this section.

### 3.1. Visual Inspection of Specimens

**Figure 8:**
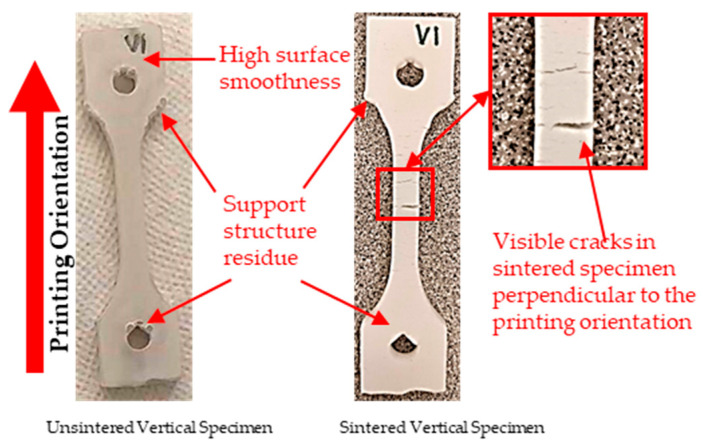
Vertical tensile specimen before and after sintering.

Figure 8 shows the unsintered and sintered vertical tensile specimens. The removal of support structures was a complex task as the material was fragile. Therefore, some support residues were still present in the testing specimens even after sintering. Regarding the surface texture of the specimens, it was observed that green specimens had smooth surfaces without visible surface cracks. In contrast, the sintered specimens had a rough surface. 

**Figure 9:**
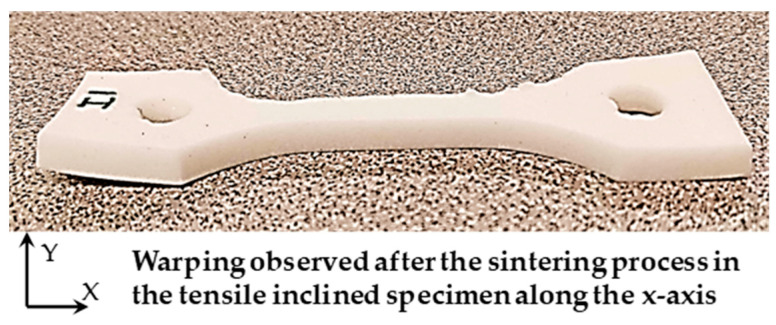
Inclined tensile specimen after sintering process.

Some visible cracks oriented perpendicular to the printing orientation were noticed after sintering the vertical samples, as shown in Figure 8. It is obvious that these cracks would significantly impact the strength of the sintered specimens. On the other hand, the sintered tensile specimens with an inclined orientation were warped, as shown in Figure 9. This would result in inaccurate strength measurement, as warped specimens induce misalignment between the tensile testing machine grips. Therefore, in this study, they were not fully tested for tensile property characterization.

### 3.2. Shrinkage Compensation Factor

The observed shrinkage in the Norton tensile specimen after sintering was used to calculate the shrinkage factor. This was used for the compensation in the modified Ford tensile test specimen before manufacturing. The aim was to acquire the desired dimensions of the specimens after sintering, as shown in Figure 2a, and to make them eligible for mechanical testing, as recommended in ISO-527-1 [29].

Table 1 shows the percentage (%) of observed shrinkage in the Norton tensile specimen. The factors were observed for overall length, shoulder width, and gauge thickness. As the gauge width is parallel to the shoulder width, the same compensation percent was used. As compression specimens are very small, a 15% compensation percentage was used for both diameter and height.

The shrinkage factor acquired from the pilot project on the Norton design was used to compensate for all the modified Ford tensile specimens, and its nominal dimensions for all the printing orientations are displayed in Table 2. These dimensions were utilized to perform the dimensional analysis while comparing the CAD model dimensions with the green specimens’ dimensions.

### 3.3. Dimensional Analysis

The dimensional analyses of the green specimens (before sintering) and the sintered specimens are detailed in this section.

#### 3.3.1. Dimensional Evaluation of Green Specimens

The dimension measurements for the tensile specimens before sintering (green specimen) are presented in Table 3, while those for the compression specimens before sintering (green specimen) are presented in Table 4. It was notable that among the tensile specimens, all the green specimens, irrespective of their printing orientations, were smaller in size in all dimensions of interest compared to the scaled CAD model. Upon evaluation of the overall length, it was found that the on-edge specimens had the least average deviation (0.44 mm) compared to the scaled CAD model. Additionally, these specimens were also consistent in length, having a very minimal range and standard deviation of 0.1 mm and 0.04 mm, respectively. The vertical specimens were next in the sequence, followed by the inclined specimens, having comparable average deviations in length of 1.32 mm and 1.57 mm, respectively. 

With regard to the shoulder width, it can be clearly seen from the table that the vertically orientated printed specimen had the lowest deviation in the negative direction and, due to its low standard deviation and range, its consistency was maintained. The on-edge and inclined specimens, on the other hand, produced very similar results for this aspect. With regard to the gauge thickness of the tensile specimens, the vertical and inclined specimens maintained their corresponding dimensions with a minimal average deviation of 0.3 mm, followed by the on-edge specimens at 0.45 mm, with an almost similar range and standard deviation.

With regard to compression (Table 4), the on-edge green specimens exhibited a lower average length (by 0.11 mm) than the scaled CAD model. However, the other two orientations of printed specimens exceeded the expected measured value by 0.02 mm for the inclined and 0.33 mm for the vertical orientation. Among all orientations, on-edge specimens had the lowest average deviation at 0.06 mm, followed by inclined specimens at 0.18 mm, and, finally, the vertical specimens at 0.26 mm. In brief, all the specimens, irrespective of their printing orientations, showed accuracy when compared to their CAD models.

#### 3.3.2. Dimensional Evaluation of Sintered Specimens

For the sintered specimens, the observed changes in the dimensions due to the evaporation of the polymeric portion and sintering of the silica ceramic part were marked. These specimens were compared to the nominal design and unsintered specimens’ dimensions, as shown in Table 5. Considering the comparison with the modified Ford tensile CAD model in terms of the overall length of the specimens, it was observed that the on-edge orientation printed specimens had the least average deviation (0.2 mm) from the standard model, followed by vertical specimens (0.4 mm) and then inclined specimens (1 mm). When the overall lengths of the green specimens were compared to the sintered ones, it was found that the lengths of vertical specimens were reduced by 11 mm upon firing, followed by inclined (8.6 mm) and then on-edge specimens (7.1 mm). The shoulder and gauge widths of the vertical specimens had the lowest range and standard deviation, while inclined and on-edge specimens had almost the same results, with an average deviation from the standard value of 1 mm for the shoulder width and around 0.8 mm for the gauge width. The gauge thickness of the inclined specimen had the least deviation of 0.04 mm when compared with the nominal design value of 5 mm. The other two dimensions had the same average value of around 4.90 mm.

Table 6 shows the dimensional variability between the recorded dimensions of the compression specimen after sintering versus the green specimens and the standard dimensions to be acquired for the testing, as per ISO 17162 [28]. The standard length of the specimen was 8 mm and the inclined specimens had an average length of 8.01 mm, which was closest to the desired value, followed by the on-edge specimens at 8.06 mm and the vertical specimens at 8.13 mm. When the green specimens were compared to the sintered ones, it was noted that the vertical specimens’ average maximum length had been reduced by around 1.45 mm during the sintering process, followed by the inclined specimens (1.21 mm) and the on-edge specimens (1.03 mm). 

Upon evaluation of the diameter, it was found that where the standard value was 9 mm, the inclined specimens and the vertical specimens exceeded the desired value by 0.05 mm and 0.12 mm, respectively. However, values for on-edge specimens were 0.24 mm less than the desired value. In this regard, the inclined specimens displayed the highest dimensional accuracy in terms of the overall specimen size. 

**Figure 10:**
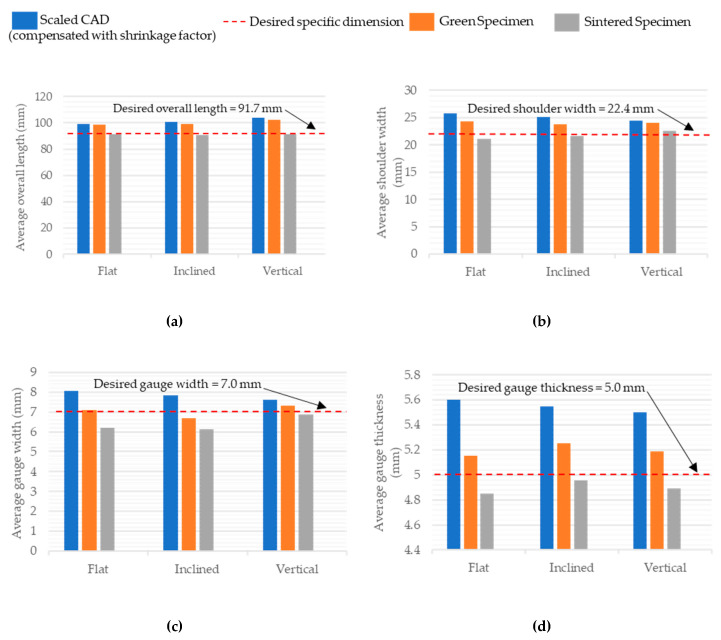
Comparison of Ford tensile specimens’ average dimensions. (**a**) Overall length, (**b**) shoulder width, (**c**) gauge width, and (**d**) gauge thickness at different stages for all the printing orientations.

**Figure 11:**
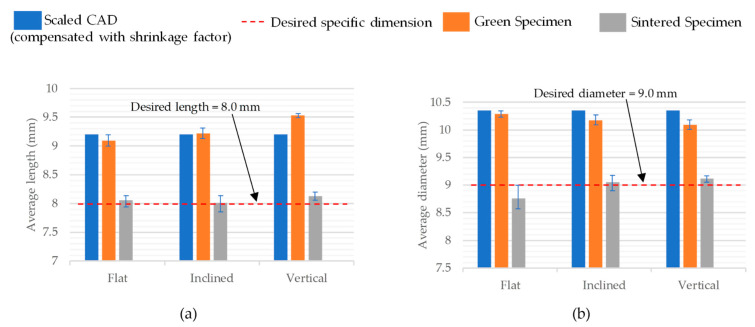
Comparison of compressive specimens’ average dimensions, (**a**) length and (**b**) diameter, at different stages for all the printing orientations.

The dimensional variations shown in Table 5 and Table 6 are graphically represented in Figure 10 and Figure 11, respectively, where the blue bar represents the dimension of the scaled CAD model, which is compensated for by the shrinkage factor. The orange bar represents the dimensions of the 3D-printed green specimens and the gray represents the sintered specimens. The red dotted line represents the desired dimension for each specimen after sintering. 

### 3.4. Mechanical Characterization

The mechanical characterization of the ceramic parts consisted of tensile and compressive tests as well as an investigation of their porosity.

#### 3.4.1. Tensile Test Results

**Figure 12:**
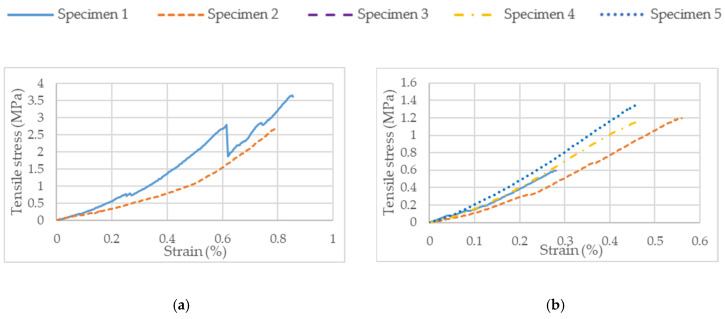
Stress–strain curve for (**a**) on-edge and (**b**) vertical tensile specimens.

After the sintering process, the inclined tensile specimens were distorted significantly with warpage. They were not suitable for the tensile testing and hence were excluded from the study. The specimens were fragile in nature and some specimens were damaged before testing. Therefore, the tensile results for the inclined specimens are not included; the stress–strain curves of the on-edge and vertical specimens are displayed in Figure 12a,b, respectively. The stress–strain curves of the tensile specimens exhibited brittle behavior typical of the silica–ceramic compound and broke directly after reaching the ultimate strength.

Table 7 depicts the tensile characteristics of the ceramic parts at different orientations. The average ultimate tensile strength of the on-edge specimen was around three times higher than that of the vertical specimen, i.e., 3 MPa. In addition to this, it is inferred that the on-edge specimens deformed twice (0.8%) compared to the vertical specimens (0.45%) before failure. Due to its brittle nature, the specimen did not possess yield strength in a practical sense, as it fractured long before reaching this point.

#### 3.4.2. Compression Test Results

**Figure 13:**
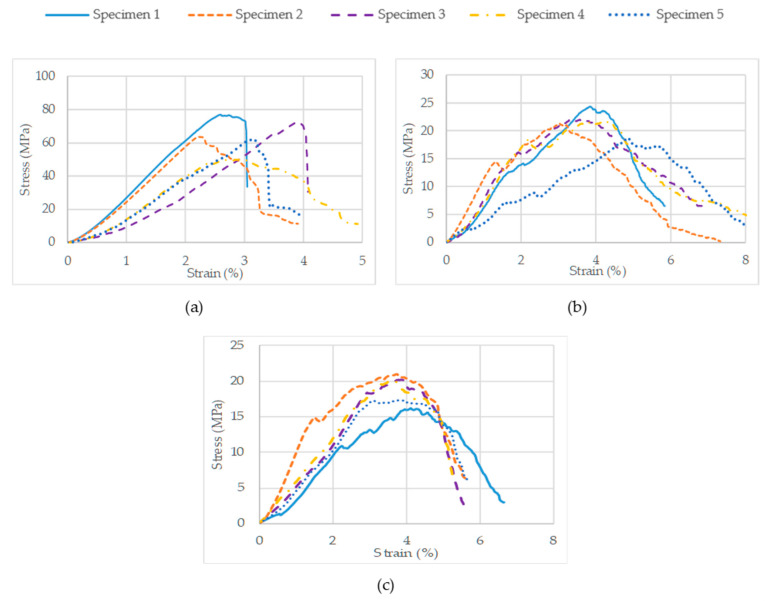
Stress–strain curves for five (**a**) on-edge, (**b**) inclined, and (**c**) vertical compression specimens.

Similarly to the tensile tests, the stress–strain curves for compression specimens are presented in Figure 13a–c for the on-edge, inclined, and vertical specimens, respectively. 

All the material characteristics of interest are tabulated in Table 8. The yield strengths of the inclined and vertical specimens were 14 and 16 MPa, respectively. However, the on-edge specimens exhibited yield strength almost four times greater, at 60 MPa. For the yield strain, it was observed that the on-edge and vertical specimens had the same average value of 2.7%, followed by the inclined specimen at 2%. Specimens manufactured in the on-edge orientation had the highest average ultimate compressive strength, at 65 MPa, followed by the inclined and vertical specimens, which had comparable ultimate compressive strength values of 22 and 19 MPa, respectively. However, in terms of the highest yield strength, the on-edge specimens had the lowest average ultimate compressive strain, at 3%, compared to the inclined and vertical specimens, which both had values of 3.9%. It can be concluded that the on-edge specimens were significantly brittle and they fractured as soon as they reached their yield strength.

### 3.5. Specimen Porosity and Weight Reduction after the Sintering Process

**Figure 14:**
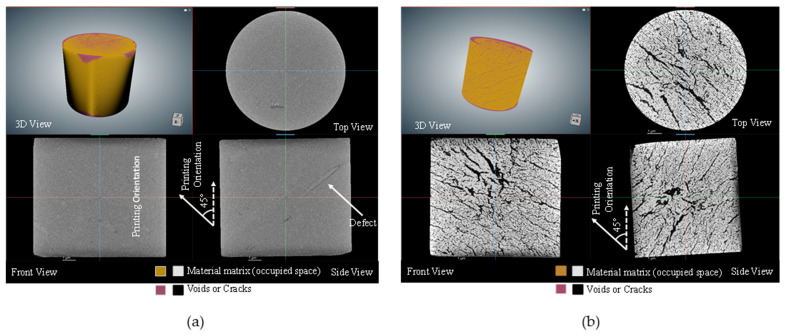
Scanned images of compression specimens, (**a**) inclined green and (**b**) inclined sintered, in 3D and three 2D sections.

**Figure 15:**
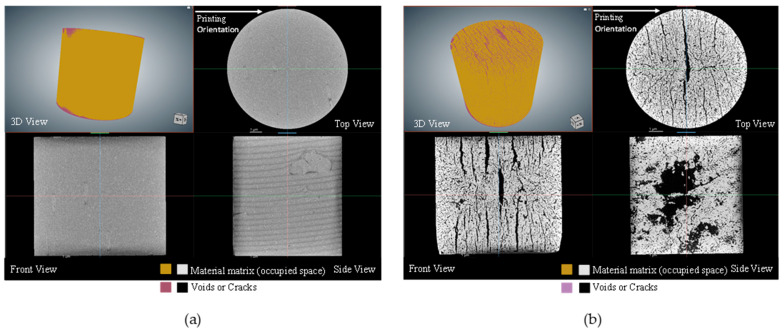
Scanned images of compression specimens, (**a**) on-edge green and (**b**) on-edge, sintered in 3D and three 2D sections.

The CT scanned data were analyzed by using the ORS-Dragonfly 2022 software. The image segmentation feature was used to differentiate between voids and the solid specimen. A pictorial comparison between the green and sintered compression specimens for both the on-edge and inclined printing orientations is shown in Figure 14 and Figure 15. Due to the presence of polymeric portions in the green specimens, porosity and cracks were not significant. However, several visible cracks and voids were observed in sintered specimens. Table 9 depicts the porosity percentages of the green and sintered compression specimens, along with the deviation in weight. For the on-edge and inclined specimens, the porosity percentage before sintering was found to be 1.26% and 1.28%, respectively. After sintering, the porosity percentage increased to 15.24% and 19.01%, respectively. Furthermore, a significant reduction in weight was also detected in the analyzed specimens. Table 9 depicts a weight reduction of nearly 30% for the same specimens after the sintering process. 

Table 10, on the other hand, illustrates the reduction in the weight along with the range that occurred in the post-sintered tensile specimens due to the sintering process. A weight reduction of approximately 33% for the tensile specimens was observed, irrespective of the print orientation. By investigating the characteristics of porosity and weight reduction, it was observed that the porosity for the sintered compression specimens varied between 15% and 19%, irrespective of the printing direction, while the weight reduction after the sintering process observed for both compression and tensile specimens ranged from around 29.5% to 33%. This demonstrates that neither parameter has a significant effect on the build orientation. Moreover, it was evident from the CT scans that the cracks were always perpendicular to the printing orientation.

It was observed that the on-edge specimen exhibited significantly higher yield strength, roughly by a factor of four. It can be seen from the CT scans that the morphology of the cracks/porosity plays a crucial role in determining the strength. In the inclined compressive specimens, the cracks were inclined at 45°, i.e., along the shear plane, which would cause shear failure. However, for the on-edge specimens, the cracks were arranged in the longitudinal direction, causing a columnar effect and lesser shear along the cracks, and, as a result, these samples had higher strength.

## 4. Conclusions

The study presented a methodology for the manufacturing of silica-based ceramic parts using stereolithography. It investigated the influence of the build orientation on the dimensional accuracy and mechanical properties due to the recognized anisotropic behavior exhibited in objects produced by SLA. Since ceramic particles are suspended in the photopolymer resin, the SLA printer produces green objects consisting of both ceramic and polymer components. These need to undergo pyrolysis to achieve pure ceramic elements. However, the sintering process results in significant shrinkage. To compensate for this shrinkage, CAD models were scaled using a shrinkage factor calculated through a pilot experiment. The scaled CAD models were then 3D printed and sintered at different orientations, and the dimensions of the final products were compared with the desired dimensions. The results demonstrated that scaling the CAD model using the compensation factor yields impressive results, with the final objects closely matching the desired dimensions, deviating by an average of approximately 3%.

On the other hand, sintering led to a 15% to 19% increase in the porosity of the specimens and a weight reduction of approximately 33% compared to the green specimens, which could potentially impact their mechanical properties. Therefore, the tensile and compressive strengths of the sintered specimens at various orientations were examined. The “on-edge” specimen exhibited the highest strength for both compression and tension.

Compared to conventional techniques, VAT polymerization shows potential in producing intricate ceramic parts. However, during this study on the impact of different orientations on the strength of ceramic stereolithography parts, some drawbacks were identified. The recommended burnout cycle, for instance, resulted in parts with cracks. Therefore, one of the future research priorities is establishing an optimized burnout cycle for this proprietary material. Additionally, investigating the influence of matrix–filler ratios on the final part’s shrinkage and strength is a crucial research avenue. Furthermore, a comprehensive analysis of ceramic part strength in relation to the microstructure should be conducted to understand the effects of cracks and porosity on the mechanical properties.

## Figures and Tables

**Table 1:** Shrinkage percentage observed in the Norton tensile specimen.

Tensile Specimen	CAD Model (Common to All Printing Orientations)	On-Edge		Inclined		Vertical	
		Sintered Specimen	Percentage Shrinkage (%)	Sintered Specimen	Percentage Shrinkage (%)	Sintered Specimen	Percentage Shrinkage (%)
Overall length (mm)	76.0	69.8	8	67.8	11	66.2	13
Shoulder width (mm)	17.1	14.5	15	15.0	12	15.5	9
Gauge thickness (mm)	5.0	4.4	12	4.43	11	4.5	10

**Table 2:** Nominal compensated dimensions in the CAD model for all print orientations (scaled CAD).

Measurement (mm)		On-Edge	Inclined	Vertical
Tensile specimen	Overall length	99.04	100.87	103.62
	Shoulder width	25.76	25.09	24.42
	Gauge width	8.05	7.84	7.63
	Gauge thickness	5.60	5.55	5.50
Compression specimen	Length	9.20	9.20	9.20
	Diameter	10.35	10.35	10.35

**Table 3:** Measured dimensions of the tensile specimen before sintering (green specimens).

Tensile Specimens		On-Edge	Inclined	Vertical
Overall length (mm)	Measured	98.6 ± 0.04	99.3 ± 0.32	102.3 ± 0.24
	α*	0.44 ± 0.04	1.57 ± 0.32	1.32 ± 0.24
Shoulder width (mm)	Measured	24.3 ± 0.57	23.73 ± 0.25	24.00 ± 0.14
	α*	1.46 ± 0.57	1.36 ± 0.25	0.42 ± 0.14
Gauge width (mm)	Measured	7.09 ± 0.25	6.7 ± 0.48	7.31 ± 0.10
	α*	0.96 ± 0.25	1.14 ± 0.48	0.32 ± 0.10
Gauge thickness (mm)	Measured	5.15 ± 0.10	5.25 ± 0.09	5.19 ± 0.11
	α*	0.45 ± 0.10	0.3 ± 0.09	0.31 ± 0.11

α* = Deviation in average measurement of the green specimen from the scaled CAD (mm).

**Table 4:** Measured dimensions of the compression specimen before sintering (green specimens).

Compression Specimens		On-Edge Average	Inclined Average	Vertical Average
Length (mm)	Measured	9.09 ± 0.11	9.22 ± 0.08	9.53 ± 0.12
	α*	0.11 ± 0.11	0.02 ± 0.08	0.33 ± 0.12
Diameter (mm)	Measured	10.29 ± 0.03	10.17 ± 0.09	10.09 ± 0.06
	α*	0.06 ± 0.03	0.18 ± 0.09	0.26 ± 0.06

α* = Deviation in average measurement of the green specimen from the scaled CAD (mm).

**Table 5:** Measured dimensions of the tensile specimen after sintering.

Tensile Specimens		On-Edge Average	Inclined Average	Vertical Average
Overall length (mm)	Measured	91.5 ± 0.35	90.7 ± 0.26	91.3 ± 0.56
	β	0.2 ± 0.35	1.0 ± 0.26	0.40 ± 0.56
	γ	7.1 ± 0.35	8.6 ± 0.26	11 ± 0.56
Shoulder width (mm)	Measured	21.18 ± 0.41	21.64 ± 0.31	22.55 ± 0.06
	β	1.22 ± 0.41	0.76 ± 0.31	0.15 ± 0.06
	γ	3.19 ± 0.41	2.09 ± 0.31	1.45 ± 0.06
Gauge width (mm)	Measured	6.19 ± 0.08	6.13 ± 0.54	6.87 ± 0.03
	β	0.81 ± 0.08	0.87 ± 0.54	0.13 ± 0.03
	γ	0.9 ± 0.08	0.57 ± 0.54	0.44 ± 0.03
Gauge thickness (mm)	Measured	4.85 ± 0.03	4.96 ± 0.10	4.89 ± 0.04
	β	0.15 ± 0.03	0.04 ± 0.10	0.11 ± 0.04
	γ	0.3 ± 0.03	0.29 ± 0.10	0.3 ± 0.04

β = Deviation in average measurement of sintered specimens from the modified Ford model (mm). γ = Deviation in average measurement of green specimen from the sintered specimen (mm).

**Table 6:** Measured dimensions of the compression specimen after sintering.

Compression Specimens		On-Edge Average	Inclined Average	Vertical Average
Length (mm)	Measured	8.06 ± 0.10	8.01 ± 0.15	8.13 ± 0.06
	β	0.06 ± 0.10	0.01 ± 0.15	0.13 ± 0.06
	γ	1.03 ± 0.10	1.21 ± 0.15	1.45 ± 0.06
Diameter (mm)	Measured	8.76 ± 0.21	9.05 ± 0.13	9.12 ± 0.03
	β	0.24 ± 0.21	0.05 ± 0.13	0.12 ± 0.03
	γ	1.53 ± 0.21	1.12 ± 0.13	1.01 ± 0.03

β = Deviation in average measurement of sintered specimens from the standard model (mm). γ = Deviation in average measurement of the green specimens from the sintered specimen (mm).

**Table 7:** Tensile specimens’ material characteristics.

Material Characteristics	On-Edge Range	Vertical Average
Ultimate tensile strength (MPa)	3 ± 0.7	1 ± 0.3
Ultimate strain (%)	0.8 ± 0.04	0.45 ± 0.1

**Table 8:** Compression specimens’ material characteristics.

Material Characteristics	On-Edge Average	Inclined Average	Vertical Average
Yield compressive strength (MPa)	60 ± 12	14 ± 3	16 ± 4
Yield strain (%)	2.7 ± 0.5	2.0 ± 0.4	2.7 ± 0.8
Ultimate compressive strength (MPa)	65 ± 10	22 ± 2	19 ± 2
Ultimate strain (%)	3.0 ± 0.6	3.9 ± 0.7	3.9 ± 0.2

**Table 9:** Increase in porosity and weight reduction observed in the compression specimens post-sintering.

Print Orientation	Characteristics	Before Sintering	After Sintering	Deviation (%)
On-edge	Weight (g)	1.32	0.93	29.54
	Range	0.02	0.03	-
	Porosity (%)	1.26	15.24	13.98
Inclined	Weight (g)	1.33	0.91	31.58
	Range	0.04	0.01	-
	Porosity (%)	1.28	19.01	17.73

**Table 10:** Weight reduction observed in the tensile specimen post-sintering.

Print Orientation	Characteristics	Before Sintering	After Sintering	Deviation (%)
On-edge	Weight (g)	14.81	9.92	33.02
	Range	1.14	0.78	-
Inclined	Weight (g)	14.05	9.47	32.60
	Range	0.94	0.66	-
Vertical	Weight (g)	14.72	9.90	32.74
	Range	0.42	0.29	-

## Data Availability

Data are contained within the article.
